# Learning space, students' collaboration, educational outcomes, and interest: Exploring the physical, social and psychological mediators

**DOI:** 10.1016/j.heliyon.2023.e15456

**Published:** 2023-04-13

**Authors:** Cecilia Obi Nja, Mary Ideba Anari, Costly Manyo Erim, Kimson Joseph Idiege, Aldeva Ilhami, Julius Ukah Ukah, Okon Eyo Eneyo, Uduak Edet Uwe, Bernedette Umali Cornelius-Ukpepi

**Affiliations:** a University of Calabar, Cross River State, Nigeria; b Universitas Islam Negeri Sultan Syarif Kasim, Pekanbaru, Riau, Indonesia

**Keywords:** Learning space, Acoustics, Psychological, Seating arrangement, Classroom environment

## Abstract

The present research article examined how the learning space correlated with students' collaboration and educational outcomes: in science students. The study investigated the foundation of psychological, social, and physical mediators that impress on students' scholarship, collaboration, and interest. The study had a sample size of 548 science students randomly selected from eleven secondary schools from a population of 985 science students in Akamkpa Local Government Area of Cross River State, Nigeria. The research design that was used in study was a cross-sectional observational type of survey. A questionnaire named Learning Space and Students Outcome Questionnaire (LPSOQ) was the tool employed in the study. The questionnaire was divided into two parts. Part A sought for student's demographic variable like age and gender. Part B had variables like physical space (seating arrangement and acoustic), psychological (self-efficacy and extrinsic motivation) and students' outcome (academic grade, collaboration and students' interest). LPSOQ reliability results ranged from 0.79 to 0.89 for Cronbach alpha and 0.81 for Kuder Richardson's formula-20. Data collected were analyzed by employing regression statistics, percentages, and mean. The regression statistics showed that the t values of seating arrangement, for academic grade (t = 5.311, p < .05), collaboration (3.627, p < .05) and interest (t = 3.463, p < .05) were statistically significant. The t values for acoustic, of academic grade (t = 4.631, p < .05), collaboration (4.020, p < .05) and interest (t = 4.631, p < .05) were statistically significant. It was recommended among others that science classroom seating arrangement should be modified to fit into the U-shape form to enable the teacher to interact freely with every student and not to be hindered by a fixed position.

## 1 Introduction

The physical environment in which learning occurs is termed a learning space [1] and can also involve the interface between a student's environment and learning [2] For effective academic processes in schools, new scientific studies suggest that the quality of students' academic outcomes is highly dependent on the function of the learning space [3]. Presently, learning approaches have shifted from teacher-centred to student-centred, so research is currently on how to increase interactive teaching that will increase collaboration [4] by adjusting the outlooks of the learning spaces [5]. Some learning spaces are specifically designed in a specific fashion to promote constructivist pedagogy [6]) at another time to enable the teacher to move from the role of a “sage on the stage” to being a “guide on the side” [7] or can be a “peer at the rear” where the instructor is involved in leading a classroom community [8]. A study conducted by Ref. [9] has indicated that learners learn more when they perceive that they are close to their teacher.

In the last decades, the relevance of classroom learning space in the shaping of how teaching and learning are undertaken has been emphasized [10]. Classroom learning space is defined as a material reality that consists of an anthropogenic environment that deals with the models of behavior of the society, humanitarian values, and actively, and dynamically controls the role of the classroom [11]. It is now a well-established fact that the terrain of teaching and learning in schools is swiftly evolving [12]. Recently, educators are required to utilize innovative techniques that will enable them to conform to the digital age to guarantee the engagement of secondary school students in the twenty-first century whilst having their learning needs. Evidence is seen in the paradigm shift in the educational sector over the last few decades from didactic teacher-led instruction to students'-centred instruction [13].

Modifications in school learning spaces have begun internationally and thereby inventing novel learning environments that foster more vigorous teaching and learning [14]. Studies by Ref. [15] indicated that when learning environments are effectively designed, there is a facilitation of the constructivist pedagogy which enhances learners' engagement. How classroom space is arranged and the aesthetic nature of the classroom is capable of influencing students' grades and changes with age and gender [16].

The student variables that are associated with their learning include some of the following demographic variables (for example; age, gender, parents' educational level, rural/urban background, etc.), students' metacognitive variables like; self-efficacy, motivation, confidence, interest, emotional states, and stress [17].

The present-day classroom is technology-infused having multiple areas for instructors. They are like U-shape student seating where flexible chairs and flexible cabinetwork have replaced the traditional classroom setting, which was made of rows of desks having just one point for the instructor [5,18]. Changes in the virtual space have brought about a paradigm shift in the educational sector where lecture-based instruction has been replaced by teamwork and viable learning method of schooling know-how [19]. Studies on the influence of learning rooms and material surroundings on classroom instructions are scanty. Bernstein's theory postulated three interconnected rules in the relationship between teachers and students: i) the superior rules, (ii) sequencing rules and pacing, [20]. With the laid down rules, students are well informed of the acceptable forms of verbal utterances, social relationships, or positions in the school [21].

The traditional features of ancient school buildings are characterized by the arrangement of the desks into rows, fixed and cannot be altered and even modern-day classrooms are arranged such that parallel rows and columns of students face the board as well as the teacher's desk. In this type of classroom arrangement, the teacher's desk is positioned close to the board. It gives the students and teachers a permanent sitting position in the classroom [22].

The self-efficacy concept has its root in Ref. [23] social learning theory. Self-efficacy is responsible for the differences that are observed in the manner in which people feel, think, and spur them. A low sense of efficacy hinders cognitive progression and achievement in diverse contexts, including how good or bad resolutions are made and academic performance [24].

**Fig. 1:**
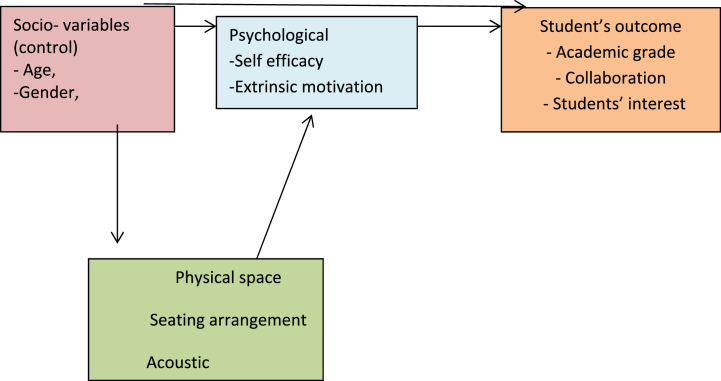
Conceptual model.

**Fig. 2:**
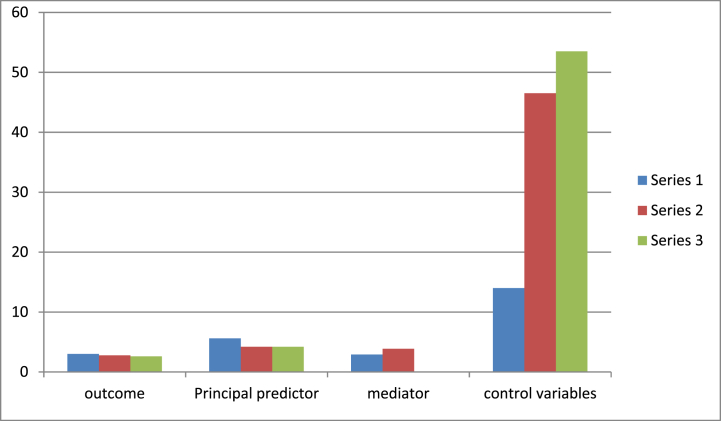
Sample descriptive graph.

The purpose of this study was to provide a thorough comprehension of how academic grades, collaboration, and interest outcomes of students are correlated with those variables that are capable of shaping classroom learning spaces. A host of mediators have been included in the study. The conceptual model that leads this empirical investigation can be seen in Fig. 1, Fig. 2.

The model has seating arrangement and acoustic factors of the physical learning space as the principal predictors. Presently, studies on classroom learning spaces have indicated increased chances for interaction among students, increased classroom discussions, more student-teacher out-of-class consultations, and improved grades and test scores, when compared to classes that are a par held in traditional seating patterns [25].

Studies reviewed provided little or no information as to the effect of the social factors (age and gender) and psychological factors (self-efficacy and extrinsic motivation) acting as mediators variables to the students' outcome (collaboration, academic grade, and students' interest). The present paper examined three learners' outcomes: academic grade, collaboration, and student interest.

It was hypothesized that physical classroom learning spaces (flexible furniture, and a better acoustic environment) benefit learners' collaboration, academic grades, and interest through self-efficacy and increased extrinsic motivation. This study expected physical classroom learning spaces to affect these outcomes both directly and indirectly through these routes and socio-demographic variables of age and gender that might bamboozle the key relationships of value. The relative vigor of the mediators' effect was not hypothesized due to scanty findings from prior work.

## 2 Methods

The study used a cross-sectional study design. The research design was chosen because it allows for the collection of data from many individuals at a single point in time where variables are only observed without influencing them [26].

The study was carried out in Calabar South, Nigeria and the performing year was 2021/2022 school year. Calabar South has eleven secondary schools and a population of 985 science students. Science students were chosen for here because of the abysmal results of science students in senior secondary school examinations [27]. The sampling technique used for this study was the hat-and-draw method.

In other to get an equivalent sample distribution through the 11 schools, 60% of the science students in each school were used. The procedure involved the writing of numbers derived from the students' attendance register on pieces of paper that were thereafter folded and dropped in a hat. A blindfolded student who was not part of the research was instructed by the research assistant to pick one folded paper in the hat at a time. Pieces of paper picked were dropped back to the hat after jotting down before any was picked to ensure that all students had equal chances of being picked. The process was repeated until 60% of the participants were picked. At the end of the exercise, 548 science students made up the sample used for the present study.

A questionnaire named Learning Space and Students Outcome Questionnaire (LPSOQ) was used as an instrument for data collection for the present study (See supplementary material.). The researchers constructed LPSOQ which was used for data collection. The instrument was divided into two parts. Part A sought students' demographic variable like age and gender (Socio-variables which was the control). Age (measured continuously in years), The sample used was made up of students aged 14 to 17 and the mean age was close to 14 years. Gender (denoted male or female) there was a perfectly balanced distribution of gender. Part B had variables like physical space (seating arrangement and acoustic), psychological (self-efficacy and extrinsic motivation), and students' outcomes (academic grade, collaboration, and student interest).

Face and content validated for LPSOQ were conducted by adepts in test and measurement and chemistry teachers. These three experts perused the questionnaire for their suitability, importance, and content of the characteristics that were being investigated, and thereafter reliability was conducted. Three items were expunged due to their appropriateness. The final number of 39 items was arrived at after eight items were revised. Students' outcome of here was made up of three sub-sections: academic grade, collaboration, and student interest. The academic grade was investigated by multiple choice questions. There were 5 questions with five options each. Every question had one correct answer and four distractors. A student can get 5 or 0 points. The LPSOQ reliability test was conducted using twenty-five science students in the Akamkpa Local Government Area of Cross River State. The essence of the test was to investigate the consistency of the instrument. The Kuder Richardson 20 formula reliability coefficient for the academic grade, from the pilot test, was 0.80 which is suitable. A reliability coefficient that ranged from 0.5 and above, is acceptable. Kuder Richardson's formula was used for the calculation of reliability because the variable was dichotomously scored [28]. The mean score was 3.00 LPSOQ section for collaboration had 5 items. It had a four-point response Likert scale of SA, A, D, where SA = 4, A = 3, D = 2, and DA = 1. It was used to access students' collaboration skills. Student scored from 5 to 20. The mean score was 2.77. The Cronbach's alpha reliability coefficient (r) for collaboration was 0.75. Students' interests also had 5 items. The mean score was 2.60, and students' interest in ‘r' was 0.84.

The psychological variable being the mediator had self-efficacy and extrinsic motivation. Aforementioned category had 5 items each on a four points Likert scale. The highest score for the psychological variable was 20 and least was 5. Self-efficacy had a mean score of 2.90 and extrinsic motivation had a mean score of 3.88. Self-efficacy and extrinsic motivation had r = 0.89 and r- 0.81 respectively.

The principal predator variable which was physical space had two categories; seating arrangement and acoustic. Both categories were made up of 7 items each on a 4 Likert scale. A student was expected to score from 7 to 28 points. The average score for seating arrangement was 5.60 and for acoustic.4.20. Seating arrangement and acoustic mean scores were above average with seating arrangement (5.60). Seating arrangement ‘r' was 0.79 and ‘r' - 0.83 for acoustic.

The researchers received permission from the schools involved in the study and the ethic of the commission of Calabar South, Teaching Board adhered to. Students who participated in this study were informed of confidentiality and anonymity concerning responses to the questionnaire. The participant willingly consented and got involved in the research. The research was conducted in the second semester of the 2021/2022 school year. Independent research assistants assisted in the administration of LPSOQ to respondents in their classes during the break period for 35 min.

The data collated from the participants (LPSOQ) were analyzed by employing descriptive and inferential statistics. Mean and percentage formed the descriptive statistics while Sobel mediating test analysis and linear regression statistics were the inferential statistics.

## 3 Results

Descriptive Statistics: The study used 3 students' outcomes; the physical space was in two-level; seating arrangement and acoustic. It was employed as the main predictors, 2 mediators, and 2 control variables.

The regression statistics in Table 1 showed that the t values of seating arrangement, for the academic grade (t = 5.311, p < .05), collaboration (3.627, p < .05) and interest (t = 3.463, p < .05) were statistically significant. Still on the regression statistics in Table 1, the t values for acoustic, of academic grade (t = 4.631, p < .05), collaboration (4.020, p < .05) and interest (t = 4.631, p < .05) were statistically significant. The regression statistics indicated the t values for the interaction between students' age and seating arrangement on academic grade was (t = 4.621, p < .05), age and collaboration (t = 4.011) were statistically significant except age with interest (t = 1.524). The t values of the regression statistics in Table 1 for the interaction between age and acoustic on students' scores (t = 4.241, p < .05), collaboration (t = 4.020, p < .05) and interest (t = 4.631, p < .05), were statistically significant. The interaction of gender with seating arrangement on interest (t = 5.028, p < .05), was statistically significant. In a similar vein, the result of the interaction of gender with acoustic on interest (t = 5.526p < .05), was statistically significant.

**Table 1:** Regression statistics of the associations between Physical classroom learning spaces and students’ outcomes.

	Seating arrangement			Acoustic		
	Academic grade	Collaboration	Interest	Academic grade	Collaboration	Interest
	Model 1	Model 2	Model 3	Model 5	Model 6	Model 7
Seating arrangement	6.765* (1.225)	6.110* (1.214)	.291* (.101)	.079* (.259)	5.733* (1.145)	.079* (.240)
	Beta = .263	Beta = .115	Beta = 141	Beta = .081	Beta = .133	Beta = .081
	t = 5.311	t = 3.627	t = 3.463	t = 2.307	t = 5.311	t = 2.307
	Sig = .000	Sig = .000	Sig = .000	Sig = .012	Sig = .000	Sig = .012
Acoustic				1.142*Sig = .012	.923*Sig = .012	1.138*Sig = .012
				(.231)	(.232)	(.241)
				Beta = .166	Beta = −.161	Beta = .166
				t = 4.631	t = 4.020	t = 4.631
				Sig = .000	Sig = .000	Sig = .000
Age	1.152* (.245)	.960* (.231)	120 (0.71)	1.142* (.245)	.948* (.234)	4.326 (3.224)
	Beta = .191	Beta = −.170	Beta = .062	Beta = .191	Beta = .162	Beta = .043
	t = 4.621	t = 4.011	t = 1.524	t = 4.241	t = 4.126	t = 1.214
	Sig = .000	Sig = .000	Sig = .100	Sig = .000	Sig = .000	Sig = .189
Gender	−.183 (.198)	1.609 (2.700)	1.239* (.246)	−.293 (.214)	1.594 (2.733)	1.321* (.226)
	Beta = .033	Beta = .026	Beta = 5.028	Beta = −.052	Beta = .026	Beta = .225
	t = −.927	t = .596	t = 5.028	t = −1.370	t = .583	t = 5.526
	Sig = −.354	Sig = .552	Sig = .000	Sig = .171	Sig = .560	Sig = .000

Sample size = 548; Coefficient presented, Standard errors in parenthesis,*p < .05.

Table 2 indicated that the t values of seating arrangement on mediating variables, of self-efficacy (t = 4.903, p < .05) and extrinsic motivation (t = 8.456, p < .05) were statistically significant. The regression statistics in Table 2 showed that the t values for the interaction between age and seating arrangement on mediating variables, of self-efficacy (t = t = 14.141, p < .05) and extrinsic motivation (t = 3.773, p < .05) were statistically significant. The interaction between gender and seating arrangement on mediating variables, of self-efficacy (t = 6.126, p < .05) and extrinsic motivation (t = 11.403, p < .05) were statistically significant. The t values of acoustic on mediating variables, of self-efficacy (t = 4.819, p < .05) and extrinsic motivation (t = 7.108, p < .05) were statistically significant. The regression statistics in Table 2 also showed that the t values of the interaction between age and acoustic on mediating variables, of self-efficacy (t = 14.078, p < .05) and extrinsic motivation (t = 4.975, p < .05) were statistically significant. The regression statistics in Table 2 also showed that the t values for the interaction between gender and acoustic on mediating variables, of self-efficacy (t = 6.102, p < .05) and extrinsic motivation were statistically significant.

**Table 2:** (A). Regression statistics of the correction between seating arrangement and mediators hypothesized. (B) Regression statistics of the relationship between acoustic and the mediators hypothesized.

	Model 1	Model 2
	Self-efficacy	Extrinsic motivation
Seating arrangement	.101* (.021)	2.058* (.243)
	Beta = .205	Beta = .340
	t = 4.903	t = 8.456
	Sig = .000	Sig = .000
Age	.284* (.020)	1.033* (.274)
	Beta = .572	Beta = .169
	t = 14.141	t = 3.773
	Sig = .000	Sig = .000
Gender	.280* (.046)	5.72* (.502)
	Beta = .267	Beta = .444
	t = 6.126	t = 11.403
	Sig = .000	Sig = .000
R squared	.042	.116
	Model 1	Model 2
	Self efficacy	Extrinsic motivation
Acoustic	.105* (.022)	1.863* (.262)
	Beta = .202	Beta = .291
	t = 4.819	t = 7.108
	Sig = .000	Sig = .000
Age	.269* (.019)	1.303* (.262)
	Beta = ,542	Beta = .214
	t = 14.078	t = 4.975
	Sig = .000	Sig = .000
Gender	.282* (.046)	6.014 (.512)
	Beta = .269	Beta = .467
	t = 6.102	t = 11.744
	Sig = .000	Sig = .000

Sample size = 548; Coefficient presented, Standard errors in parenthesis,*p < .05.

The regression statistics in Table 3 showed that the t values of self-efficacy, for the academic grade (t = 4.493, p < .05), collaboration (t = 4.946, p < .05), and interest (t = 9.769, p < .05), were statistically significant. The regression statistics in Table 3 showed that the t values of extrinsic motivation, for the academic grade (t = 5.705, p < .05) collaboration (t = 3.653, p < .05), and interest (t = 9.508, p < .05), were statistically significant. Table 3 also indicated that the t values of gender, for the academic grade (t = 6.399, p < .05) collaboration (t = 3.015, p < .05), and interest (t = 6.010, p < .05), were statistically significant.

**Table 3:** Regression statistics of the relationship between the hypothesized mediators and outcomes.

Mediators	Academic grade	Collaboration	Interest
Self-efficacy	1.005* (.224)	.759* (.153)	15.494 (1.586)
	Beta = .189	Beta = .207	Beta = .386
	t = 4.493	t = 4.946	t = 9.769
	Sig = .000	Sig = .000	Sig = .000
Extrinsic motivation	.103* (.018)	.189* (.052)	1.311* (.138)
	Beta = .237	Beta = .154	Beta = .377
	t = 5.705	t = 3.653	t = 9.508
	Sig = .000	Sig = .000	Sig = .000
Control			
Age	.886* (.315)	.430 (3.476)	.183 (.314)
	Beta = .120	Beta = .005	Beta = .025
	t = 2.815	t = .124	t = .583
	Sig = .006	Sig = .902	Sig = .560
Gender	1.473* (.230)	7.820* (2.594)	1.378* (.229)
	Beta = .264	Beta = .128	Beta = .249
	t = 6.399	t = 3.015	t = 6.010
	Sig = .000	Sig = .000	Sig = .000

Sample size = 548; Coefficient presented, Standard errors in parenthesis,*p < .05.

### 3.1 Mediators' impact

Table 4 indicated that when moderating the autonomous variable (physical space), the mediating variable (self-efficacy and extrinsic motivation) and control (gender) which were t = 5.228, p < .000, t = 4.723, p < .000, t = 8.533, p < .000; t = 5.705, p < .000, t = 4.730, p < .000, t = 4.946, p < .000; t = 6.399, p < .000, t = 3.015, p < .000 and t = 6.010, p < .000 significantly predicted the dependent variable (academic grade, collaboration, and interest) respectively. When seating arrangement was controlled in Table 4, the mediating variables self-efficacy, extrinsic motivation, and gender significantly predicted the dependent variables of academic grade collaboration and interest. Mediating variables were carried out singly through the Sobel mediation test as shown in Table 5.

**Table 4:** Regression statistics of the mediating effects.

	School Grades	Collaboration	interest
	Model 1	Model 2	Model 3
Seating arrangement	.623* (.119)	.587* (.124)	.258* (.030)
	Beta = .179	Beta = .158	Beta = .343
	t = 5.228	t = 4.723	t = 8.533
	Sig = .000	Sig = .000	Sig = .000
Self-efficacy	1.005* (.224)	1.171* (.248)	.743* (.108)
	Beta = .189	Beta = .198	Beta = .281
	t = 4.493	t = 4.730	t = 6.852
	Sig = .000	Sig = .000	Sig = .000
Extrinsic motivation	.103* (.018)	1.311* (.138)	.759* (.153)
	Beta = .237	Beta = .377	Beta = .207
	t = 5.705	t = 9.508	t = 4.946
	Sig = .000	Sig = .000	Sig = .000
Age	886 (.315)	.183 (.314)	.183 (.314)
	Beta = .120	Beta = .025	Beta = .025
	t = 2.815	t = .583	t = .583
	Sig = .006	Sig = .560	Sig = .560
Gender	1.473* (.230)	7.820* (2.594)	1.378* (.229)
	Beta = .264	Beta = .128	Beta = .249
	t = 6.399	t = 3.015	t = 6.010
	Sig = .000	Sig = .000	Sig = .000

Sample size = 548; Coefficient presented, Standard errors in parenthesis,*p < .0.

**Table 5:** Proportions of total effect mediated.

Mediators	Academic grade	collaboration	interest
Self –efficacy	44.7%	40.58%	56.20%
Extrinsic motivation	48.25%	55.30%	33.10%
Gender	35.00%	34.56%	49.35%

Sobel mediating test findings as recorded in Table 5 indicated the indirect impact of self-efficacy as being strongest of the total effect of seating arrangement on interest (56.20%). Extrinsic motivation significantly mediated between seating arrangement and collaboration by 55.30%. Gender was the mediator with the largest indirect effect on academic grades (48.00%).

## 4 Discussion

The physical environment in which learning occurs and the interface between a student's environment and learning is termed, a learning space [[29], [30], [31], [32], [33]]. For effective academic processes in schools, new scientific studies suggest that the quality of students' academic outcomes is highly dependent on the function of the learning space [[34], [35], [36], [37], [38]].

Presently, studies on U-shape classroom learning spaces have indicated increased chances for interaction among students, increased classroom discussions, more student-teacher out-of-class consultations, and improved grades and test scores, when compared to classes that are a par held in traditional seating patterns [[39], [40], [41], [42], [43], [44]].

Some studies have indicated that traditional seating pattern is effective in getting learners' engagement in the classroom [45].

It was hypothesized that physical classroom learning spaces (flexible furniture, and a better acoustic environment) benefit learners' collaboration, academic grades, and interest through self-efficacy and increased extrinsic motivation. The present study expected physical classroom learning spaces to affect these outcomes both directly and indirectly through these routes and socio-demographic variables of age and gender that might bamboozle the key relationships of value. The relative vigor of the mediators' effect was not hypothesized due to scanty findings from prior work.

The influence of the physical learning space on science students' outcomes was examined. The research was conducted in Cross River State, Nigeria. Results obtained here after analysis agreed with findings from researchers outside the shores of Nigeria. Researchers in Zealand and China conducted studies on learning spaces and attested to their relevance of it. Findings obtained showed the importance of seating arrangement and acoustics on students' outcomes. Students' outcomes investigated here were; academic grade, collaboration, and interest.

Results obtained also indicated the influence of mediators (self-efficacy and extrinsic motivation) on learners' outcomes (academic grade, collaboration, and interest). Furthermore, the impact of extrinsic motivation (mediating variable) appears to be very germane in learners' academic grades.

From the results obtained, the importance of checking for mediators' variables in the flexible classroom which is capable of influencing the results of research should be encouraged. The present study advocates for perpetual developmental research to be conducted extensively to control for mediating variables. The essence is to present the amplification of some variables. Reports showed that seating arrangement affected the smooth functioning of the teaching and learning system. The flexible seating arrangement provided every student with the opportunity to have an unobstructed view of the instructional material and so encouraged learners' outcomes. In collaboration with this study [46,47,48,48,49]. [49]; indicated that seating arrangement is capable of determining the academic performance of students. Seating arrangement can influence the academic performance of students because engagement of students is achieved through seating arrangement in the classroom [50,51] Research agreed here as the researcher advocated that effective classroom layout gives rise to the desired learning outcome. In a classroom that mandates students to face the teacher, and has a screen or chalkboard located at the center, learners in front will not be able to view those at the rear, and as such collaboration in such a classroom will be hindered. Visibility will be poor and active class engagement will be lacking [[52], [53], [54]].

The type of seating arrangement in the classroom will determine the types of activities that go on in the classroom. The flexible classroom where students sit in groups brings about interaction with their peers and therefore acceptability. Having the psychological feeling that you are a member of a community is an important precursor to a robust learning outcome [[55], [56], [57], [58]].

Self-efficacy and extrinsic motivation of students mediated learners' academic grades, collaboration, and interest in this study. If a student believed she/he can pass an examination that student can pass that particular examination [[59], [60], [61]]. [62] stated that self-efficacy has been discovered as a potent force that directs students' performance. A study by Refs. [[63], [64],^65^] indicated that students with low self-efficacy feel shy to ask for assistance when they need help like their counterparts with high self-efficacy that can easily ask for help when they need it thereby making them excel in school.

The classroom layout can therefore determine learners' behaviors and motivations as collaborated by Ref. [66]. The result was so because students who work in groups in flexible seating arrangement develops high self-efficacy as a result of extrinsic motivation in a flexible classroom.

Findings indicated a significant impact of acoustics on students' collaboration. The importance of sound quality during collaboration would have been responsible for such a result. Collaboration in the classroom can only be effective when students hear themselves. Good sound quality reduces stress in students thereby increasing interpersonal relationships which enhances collaboration [67,68]. Agreed with the present study as their research emphasized that the acoustics factor in the learning space it's a recognizable impact on the subjective and learner outcomes. Other studies by Refs. [[69], [70], [71], [72]] indicated that the presence of noise in the classroom was found to hinder learning. The interpretation of the result is that seating arrangement gives particular attention to the physical space merits; acoustics takes care of the temperature of the learning space. It is connected to the emotional satisfaction or deprivation of sound. A study on classroom learning space in terms of acoustic indicated that about 25% of students' outcome was dependent on the sound quality in the classroom [34].

Extrinsic motivation exhibited the greatest mediator influence on collaboration which was followed by self-efficacy. Higher values in the extrinsic motivation variable indicated good external motivation arising from the physical learning space. The extrinsic motivation directly enhanced students; collaboration development and educational outcome. The theories that emphasized the important role of students ‘independence and leading their learning'' in playing a part in children learning were collaborated by this finding [45]. Hence, classroom space in terms of seating arrangement enabled students to move freely in the class, and have a sense of belongings as students sit in groups and the sound quality is such that they can hear themselves clearly without hindrances.

The study indicated that flexible seating arrangement type and good quality sound positively and significantly related to higher extrinsic motivation, yet the route of joining physical learning space to extrinsic motivation is not known. Further study should focus on investigating the predictors of students' outcomes like; intrinsic motivation, emotional motivation, and self-concept to supply proof of the seating arrangement, colour of the classroom, and acoustics impact on students' learning outcomes. There is also the need for a study on perpetual developmental research to be conducted extensively to control for mediating variables.

Due to the nature of the cross-sectional observational study design, it was difficult to derive causal relationships from these findings. The measures used were all based on self-reports, which are inevitably subjected to response bias due to issues such as participants' memory loss and information bias due to social desirability tendencies. Apart from memory loss, the learning outcome of students may not be only linked to learning space but other variables like teaching methods, and teachers' characteristics among others. The topic in chemistry that was used to test students understanding in the learning space may have affected the results. Teacher variables can also limit the result because teachers had varying levels of experience or training and technology usage, this could affect the reliability of the data collected.

## 5 Conclusion

The report obtained indicated that the academic achievement and collaboration skills of science students were enhanced by U-shape seating arrangements in the classroom. The present research also showed a significant effect of a good acoustic environment on students' academic achievement and collaboration. The controlled effect of gender was strong when a pod-style seating arrangement was used in a science classroom. It indirectly influenced academic achievement as well as students' collaboration skills. The mediating effect extrinsic variable when pod-style seating arrangement was used in the classroom also affected students learning outcomes as the seating arrangement motivated students to learn. The U-shape seating arrangement enables student-student interaction.

## 6 Recommendation

The interaction in the classroom motivated students to learn as such there was improved academic achievement and collaboration skills of science students. In place of the findings, the following recommendations were made;

Science classroom seating arrangement should be modified to fit into the U-shape form to enable the teacher to interact freely with every student and not to be hindered by a fixed position in the classroom. Science classrooms should have a good acoustic environment that will enable students to hear their teachers.

## Author contribution statement

Dr NJA, CECILIA OBI: Conceived and designed the experiments; Analyzed and interpreted the data; Wrote the paper.

ANARI, MARY IDEBA: Conceived and designed the experiments; Wrote the paper.

Dr. ERIM, COSTLY MANYO; Dr. UWE, UDUAK EDET; Prof. CORNELIUS-UKPEPI, BERNEDETTE. UMALI: Analyzed and interpreted the data; Wrote the paper.

Dr. IDIEGE, KIMSON JOSEPH; ILHAMI, ALDEVA; Dr. UKAH, JULIUS UKAH: Contributed materials, analysis tools or data; Wrote the paper.

Dr. ENEYO, OKON EYO: Performed the experiments; Wrote the paper.

## Data availability statement

Data will be made available on request.

## Declaration of interest's statement

The authors declare no conflict of interest.

## Additional information

Supplementary content related to this article has been published online at [URL].
