# Supplementary material for: Learning space, students' collaboration, educational outcomes, and interest: Exploring the physical, social and psychological mediators
Source: Heliyon. 2023 Apr 13;9(4):e15456. doi: 10.1016/j.heliyon.2023.e15456 (PMC10131041; doi:10.1016/j.heliyon.2023.e15456)
Supplement: Multimedia component 1 [file mmc1.docx]

Supplementary material Learning Space and Students Outcome Questionnaire (LPSOQ)

Part A ; Students’ demographic data (tick the appropriate answer)

Age: 14, 15, 16,, 17

Gender: Male Female

Part B: SA = Strongly agreed, A = Agreed, D= disagreed and SD= Strongly Disagreed

Academic grade: (tick the appropriate answer)

1. What is the oxidation number of chromium in K_2_CrO_4_?

a) +1

b) +2

c) +4

d) +6

e) + 5

2. An atom of an element X gains two electrons. The symbol of the ion formed is

a) X+

b) X^2+^

c) X^2-^

d) X

e) 2X

3. During balancing of redox reaction in acidic medium, ——————— is added

a) Hydrogen ion and water

b) Hydrogen ion and hydroxyl ion

c) Water and oxygen

d) Water and hydroxyl ion

e) Oxygen and hydrogen

4. Reduction is the process of

a) Loss of electron

b) Loss of hydrogen

c) Loss of oxygen

d) Addition of electromagnetic elements

e) increase in oxidation number

5. In the reaction CH_4_ +2O_2_ ———> CO_2_ +2H2O, what is the oxidation number of carbon in CH4

a) +4

b) -4

c) +2

d) -2

e) +1

**COLLABORATION**

Tick the correct option to the following statements. SA A D SD

1. I seek the opinions of people when confronted with a task needed action by me.

2. Working in a group to solve a problem makes me excited

3. I enjoy collaboration as it enables me to ask my group mates questions about what does not seem clear to me.

4. I like to work with others because it enables me to accomplish my task very fast.

5. I see the need for collaboration in the classroom

**INTEREST**

Tick the correct option to the following statements. SA A D SD

1. I like chemistry course more than the others.

2. I enjoy chemistry exercises even if it is every day.

3. I will like to explain concepts in chemistry to my classmates

4. Information gained during chemistry classes will not be futile after my graduation.

5. I practice chemistry outside the classroom

Self-efficacy

Tick the correct option to the following statements. SA A D SD

1.I am sure that I possess the required ability to pass the chemistry exams

with high grade.

2. I am sure that I get involve all practical exercise in the chemistry laboratory.

3. I am sure that I can give a helping hand to classmate when they have difficulties with chemistry concepts

4. I am sure that I can carry out chemistry practical during examination following instructions given.

5. I am sure of studying chemistry beyond the secondary school level.

**Extrinsic motivation**

Tick the correct option to the following statements. SA A D SD

1. I have so many great ideas, if only I could get motivated.
2. I do well with deadlines, but I can't seem to motivate myself.
3. I can't seem to do well in school unless I have someone constantly checking on me.
4. I can only read when I see my classmate reading.
5. When I see someone receiving award I work hard to have the award too.

**Physical space**

ACOUSTIC QUESTIONNAIRE

Tick the correct option to the following statements. SA A D SD

1. The overall classroom listening environment is good
2. I have a problems with the noise created inside the classroom
3. The noise generated inside the classroom by student is enormous.
4. I do not have problems with outside noise entering my classroom
5. The noise inside the classroom is as a result of echo in the classroom.
6. From where the teacher is standing in the classroom I seem to have difficulty hearing
7. My classroom is noise proof.

SEATING ARRANGEMENT

Tick the correct option to the following statements. SA A D SD

1. My teacher seating position is usually in front of the classroom
2. My teacher seating position is usually at the center of the classroom
3. My seating position is usually in front of the classroom
4. My seating position is usually at the back of the classroom
5. The seating position in our classroom is u-shape
6. In my classroom seats are movable
7. In my classroom we can form small sitting groups
